# Selection Criteria of Cord Blood Units for Platelet Gel Production: Proposed Directions from Hellenic Cord Blood Bank. Comment on Mallis et al. Short Term Results of Fibrin Gel Obtained from Cord Blood Units: A Preliminary in Vitro Study. *Bioengineering* 2019, *6*, 66

**DOI:** 10.3390/bioengineering8050053

**Published:** 2021-04-27

**Authors:** Panagiotis Mallis, Efstathios Michalopoulos, Effrosyni Panagouli, Zetta Dimou, Eirini Faidra Sarri, Eleni Georgiou, Vasiliki Gkioka, Catherine Stavropoulos-Giokas

**Affiliations:** Hellenic Cord Blood Bank, Biomedical Research Foundation Academy of Athens, 4 Soranou Ephessiou Street, 115 27 Athens, Greece; smichal@bioacademy.gr (E.M.); efi.panagouli.bio@gmail.com (E.P.); zdimou@bioacademy.gr (Z.D.); sarri.fe@gmail.com (E.F.S.); georgiou.eleni86@gmail.com (E.G.); vgkioka@gmail.com (V.G.); cstavrop@bioacademy.gr (C.S.-G.)

**Keywords:** cord blood platelet gel, wound healing, platelets, criteria, protein content, growth factors

## Abstract

This article provides additional knowledge for cord blood platelet gel (CBPG) production. Recently, it has been shown that CBPG exerts beneficial properties in wound healing applications. CBPG is produced after a two-step centrifugation process, following the addition of calcium gluconate. Clinical-grade CBPG can be produced in public cord blood banks, worldwide. However, standardization of the CBPG production process must be established in order to reduce discrepancies that occurred due to different platelet gel preparations. This article aims to provide an update regarding the selection criteria of cord blood units (CBUs), and to provide evidence for the improvement of the CBPG production process. (Comment on “Short Term Results of Fibrin Gel Obtained from Cord Blood Units: A Preliminary in Vitro Study” *Bioengineering* **2019**, *6*, 66).

Dear Editor,

The content of this article concerns the optimization and standardization of the platelet gel (PG) manual production process which utilizes cord blood (CB). Currently, there exists enough evidence to prove the beneficial effects of CBPG in wound healing applications [1,2]. Wound healing is a dynamic process of cellular events resulting in proper tissue regeneration and skin closure. In this process, specific cellular populations such as epithelial, endothelial, fibroblasts and perivascular cells are mobilized to the wounded site [3]. Besides that, the occurred inflammation, caused by mechanical injury, promotes the activation of immune cells, such as macrophages, T and B cells [4]. Immune cells have a significant role in the overall wound healing process, either by preventing the entrance of pathogens or by secreting inflammation mediators, such as growth factors and pro-inflammatory cytokines (IL-1α, IL-6, TNF-α, etc.) [4]. Based on the previous literature, CBPG may potentially accelerate and improve the wound healing process [1,2,3,4,5,6]. CBPG was used for the first time by Rebulla et al. [1,5,6] in pediatric patients suffering from extensive skin erosions caused by Epidermolysis bullosa. Since then, CBPG has been applied with great success in a great number of clinical applications, including spinal reflex recovery [7], reconstruction of deep surgical sites after cardiothoracic surgery [8], and diabetic foot ulcer healing [9]. Moreover, new methods for efficient platelet gel production, such as automatic and semi-automatic, using commercially available kits and dedicated instruments, are currently being evaluated. However, the results remain controversial, while the production cost of automated methods is higher compared to manual processes [10]. The beneficial properties of CBPG can be explained by its high-value protein content. The CBPG protein content is enriched in growth factors such as TGF-β, VEGF, ΙGF, PDGF, cytokines such as IL-1, IL-6. IL-10, TNF-α, and chemokines such as CCL3, CCL5 [11,12]. Furthermore, Rebulla et al. [13] proposed the production of CBPG from Cord Blood Units (CBUs) that do not meet the criteria for hemopoietic stem cell (HSC) isolation, banking, and release for transplantation, as defined by the Foundation for the Accreditation of Cellular Therapy—NetCord (FACT-NetCord) standards [14]. The initial volume of CBUs plays a significant role in the resulting platelet yield of CBPG. Indeed, high volume CBUs (111–148 mL) have a greater number of platelets (PLTs) in comparison to low volume CBUs (<81 mL). On the other hand, an average of 80% of the CBUs that are delivered to public cord blood banks globally are characterized either by low HSC content or by low volume [15]. Therefore, the proper production of CBPG (within the published acceptance criteria released by Rebulla et al.) from a single low volume CBU is a demanding task which, most of the time, can lead to failure [13]. In this sense, and based on previous results from our lab [16], we have shown the efficient production of CBPG, utilizing low volume CBUs. This article aims to provide an update regarding the selection criteria of CBUs to produce CBPG with a high platelet content. In this way, CBUs with a volume of <81 mL (including the anticoagulant) can be utilized to produce CBPG with a high platelet content. Briefly, PG is produced after a two-step centrifugation process of the CBUs (soft spin, 210 g for 15 min and hard spin, 2600 g for 20 min) that results in platelet-rich plasma (PRP) production. Once PRP has been produced, it can be stored at −80 °C, until further use. When CPBG is required, rapid thawing of the stored PRP, followed by filtration through a 0.22 μm filter, is performed. Then, the addition of calcium gluconate in thawed PRP (1 part of calcium gluconate to 3 parts of PRP), and incubation for 10–15 min at 37 °C, leads to CBPG formation. It should also be stated that all CBUs, prior to performing the CBPG process, must be followed by signed informed consent from the mothers. The initial steps in the CBPG production process involve cell enumeration (with a hematological analyzer) and volume determination of the CBUs. CBUs are suitable for CBPG production when white blood cells concentration (WBCs) is <12 × 10^6^/mL and PLTs concentration is >150 × 10^6^/mL [9]. However, CBUs with lower platelet concentration may also be suitable for CBPG production. Besides the aforementioned acceptable endpoints, an additional criterion must be introduced. Therefore, the initial platelet number must be determined and strongly be taken into consideration for the selection of CBUs. It has been shown that CBUs with a PLT number of >13,000 × 10^6^ can also efficiently be utilized for CBPG development (Table 1) [12]. Indeed, CBUs that do not fulfill the platelet concentration endpoint (due to initial CBU volume variance) may have acceptable platelet numbers, and therefore cannot be rejected and used as starting material for CBPG production. In addition, low volume CBUs (55–81 mL) can be pooled based on their ABO/Rh compatibility, resulting in higher volume CBUs (111–148 mL) development (Table 1). From results coming from our lab, it has been shown that pooled CBUs may have a PLT concentration comparable to an equal volume single CBU [12]. Specifically, CBPG obtained from pooled CBUs were characterized by similar platelet recovery rates (both in concentration and number), final volume, and developed fibrin gel area, compared to an equal volume single CBU. The same study also showed that concentrations of white and red blood cells (RBCs) were low (WBCs < 4 × 10^6^/mL, RBCs: <0.1 × 10^9^/mL), and that no significant variation existed between CBPGs produced either by the pooled CBUs or by an equal volume single CBU [12]. Finally, the platelet number should also be taken into account. CBPG with platelet numbers 4000–18,000 × 10^6^ are acceptable and can exert their therapeutic beneficial properties in wound healing applications (Table 2). It should also be clearly stated that all CBPG products before human application ζ must be negative for infectious diseases, including HIV, HBV, HGV, HTLV-I/II, CMV, HCV, HAV, WNV, T Pallidum, syphilis, mycoplasma, aerobic and anaerobic bacteria (Table 2). The criteria presented in this article may provide additional knowledge for the efficient production of CBPG. Medium (82–109 mL) and high (110–148 mL) volume CBUs are characterized by higher HSC content compared to those with low volume (<82 mL). CBUs that are not suitable for HSC isolation and banking are no longer rejected; therefore, they can be utilized for CBPG development. In this way, low volume CBUs can be pooled in order to form units with higher volume which are eligible for platelet gel production. All CBPG products intended for human use must be accompanied by a certificate of analysis that can be provided both to the patient and the health care professionals. Therefore, the clinical utility of CBPG may be better ascertained, as bias due to endpoints’ variability (mainly in PLTs concentration and number) of CBPG products is reduced. Health care authorities such as national blood banks and public cord blood banks (FACT-NetCord Accredited) can produce high quality clinical-grade CBPG products. The produced CBPG can serve as a useful tool for physicians in wound healing and regenerative medicine applications.

## Figures and Tables

**Table 1:** Acceptance criteria of CBUs prior to CBPG processing.

Parameter	Acceptable Endpoints
CBU volume (including the anticoagulant volume)	≥55 mL *
WBCs	<12 × 10^6^/mL
PLTs concentration	≥150 × 10^6^/mL
PLTs number	≥13,000 × 10^6^

* Low volume CBUs (55–81 mL) can be pooled based on their blood group compatibility.

**Table 2:** Acceptance criteria of CBPG.

Parameter	Acceptable Endpoints
Platelet gel volume	5–15 mL
WBCs concentration	<4 × 10^6^/mL
RBCs concentration	<0.1 × 10^9^/mL
PLTs concentration	800–1200 × 10^6^/mL
PLTs number	4000–18,000 × 10^6^
Infectious Ddsease Agents *	Negative
Microbial control **	Negative

* Infectious disease agents include testing for HIV, HBV, HGV, HTLV-I/II, CMV, HCV, HAV, WNV, T Pallidum, and syphilis. ** Microbial control includes the testing for mycoplasma, aerobic and anaerobic bacteria.

## Data Availability

Supporting data can be found in the already published manuscript “Short Term Results of Fibrin Gel Obtained from Cord Blood Units: A Preliminary in Vitro Study” Bioengineering 2019, 6, 66, doi: 10.3390/bioengineering6030066, https://www.mdpi.com/2306-5354/6/3/66.”
